# Kidney Protection, Obesity Treatment, and Multiorgan Biology: When More Therapy Is—And Is Not—Better

**DOI:** 10.1111/1753-0407.70284

**Published:** 2026-10-20

**Authors:** Zachary T. Bloomgarden, Yehuda Handelsman

**Affiliations:** ^1^ Division of Endocrinology, Diabetes and Bone Disease, Department of Medicine Icahn School of Medicine at Mount Sinai New York New York USA; ^2^ The Metabolic Institute of America Tarzana California USA

## 1 A Meeting Report and Critical Synthesis—Not a Clinical Practice Guideline

The first commentary in this series considered why HiD is best understood as a systems‐oriented care model rather than a new organ‐based specialty. This second commentary focuses on two areas in which that model is especially important: chronic kidney disease (CKD) and obesity. The kidney sessions emphasized multi‐mechanism protection and the proper interpretation of the early eGFR dip; the obesity sessions shifted attention from the amount of weight lost toward the quality, durability, and functional consequences of weight loss.

Across both areas, the central caution is similar. A plausible mechanism, a biomarker change, a DEXA finding, or a projected additive effect is not the same as an outcomes trial. New therapies should be welcomed when they add clinically meaningful benefit, but novelty alone should not displace effective, less expensive, and better‐established approaches.

## 2 Kidney Disease: From Sequential Escalation to Coordinated Protection

### 2.1 The Scale of the Problem and the Detection Failure

In a session on CKD, its frequency, lack of recognition in much clinical practice, and greater association with cardiovascular death than with kidney failure were discussed along with the important implication that to appropriately screen for CKD requires both estimated glomerular filtration rate (eGFR) and urine albumin‐to‐creatinine ratio (UACR) measurement. Neither test is sufficient alone, and neither should be treated as a one‐time measurement. The KDIGO heat map is valuable because it links falling GFR and rising albuminuria to kidney failure, heart failure, atherosclerotic events, arrhythmia and mortality, and equally because the heat map shows that the greatest population burden of CKD is among individuals with relatively less marked abnormality in eGFR and in UACR (Figure 1) [1].

**FIGURE 1:**
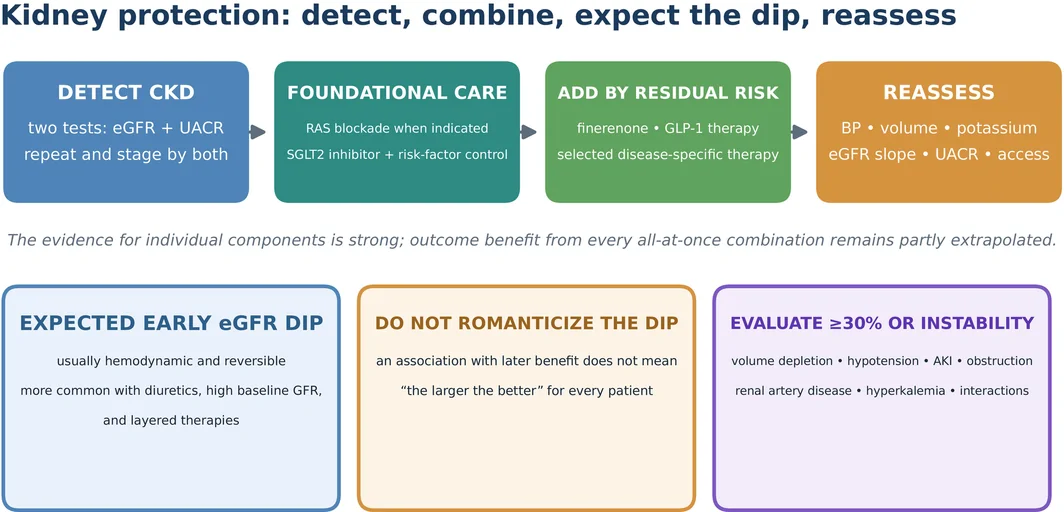
CKD care: Two‐test detection, early foundational treatment, risk‐based additions, and contextual interpretation of the eGFR dip.

The meeting highlighted a striking implementation gap. Even with established evidence of kidney and cardiovascular benefits of sodium‐glucose transport protein 2 inhibitors (SGLT2i) and the nonsteroidal mineralocorticoid antagonist (nsMRA) finerenone, including a synergistic benefit when prescribing an initial combination of SGLT2i and nsMRA, persistent real‐world use in eligible CKD and diabetes populations remains low. The urgent problem is not only discovery. It is finding CKD, prescribing evidence‐based therapy, monitoring it and keeping patients on treatment.

### 2.2 The Multi‐Mechanism Treatment Model

Renin‐angiotensin‐aldosterone system (RAAS) blockade, SGLT2 inhibition, finerenone, and glucagon‐like peptide (GLP)‐1–based therapy target overlapping but nonidentical pathways. RAAS blockade reduces intraglomerular pressure and proteinuria; SGLT2 inhibition modifies tubuloglomerular feedback, volume, and heart‐failure risk; nsMRAs target mineralocorticoid‐receptor–mediated inflammation and fibrosis; and semaglutide has now demonstrated kidney, cardiovascular, and survival benefit in T2D and CKD in FLOW [2, 3, 4, 5, 6, 7, 8]. CONFIDENCE showed greater albuminuria reduction with initial finerenone plus empagliflozin than with either alone, supporting biological complementarity [3].

The word “pillar” is useful for implementation but can imply stronger direct evidence than exists. Each component has substantial trial support in defined populations; but we must recognize that the full four‐component package has not been tested in a single outcomes trial against sequential or individualized care. Actuarial estimates of a 50%–60% reduction in kidney progression with combined therapy represent projections, not randomized controlled trial proof. The more responsible framing is to use appropriate risk‐based implementation with close reassessment rather than indiscriminate simultaneous prescribing.

Guideline status also requires precision. At the time of the meeting, the KDIGO 2024 CKD guideline remained the current global standard, while the KDIGO 2026 Diabetes and CKD update was available as a public‐review draft rather than a final guideline [1, 9]. The recommendation for simultaneous initiation may anticipate the direction of guidance, but the draft report is not a finalized 2026 standard.

### 2.3 The eGFR Dip: Expected Physiology, Not a Ritual of Reassurance

An acute fall in eGFR after starting RAAS blockade or an SGLT2 inhibitor often reflects a hemodynamic reduction in intraglomerular pressure rather than structural kidney injury. Similar, usually smaller, changes occur with finerenone and GLP‐1 therapy, and combination therapy can produce additive early declines. The key clinical insight is that an eGFR dip should be anticipated, interpreted in context and not used reflexively as a reason to stop a disease‐modifying drug [1, 3, 10].

Although the phrase “the larger the dip, the better the outcome” is attractive, it must be recognized as being overly simplistic. Observational associations between an early dip and a slower later slope can reflect therapeutic engagement, but they do not make a larger fall desirable in every patient. A decline of 30% or more exceeds expected variability and warrants evaluation for volume depletion, hypotension, acute kidney injury, obstruction, renal artery disease, hyperkalemia, excessive diuresis, or interacting drugs [1]. Symptoms and hemodynamic stability matter more than a slogan.

### 2.4 What Remains Uncertain

The presence of GLP‐1 receptors in kidney and immune cells is provocative, and emerging human mechanistic work may support a direct renal effect of GLP‐1RA. The FLOW study showed clinical benefit but did not by itself establish which cellular mechanism caused that benefit. Similarly, endothelin receptor antagonists may become useful in selected non‐diabetic kidney diseases or with persistent proteinuria, but edema and heart‐failure risk remain major constraints. Precision nephrology based on biopsy, transcriptomics and disease‐specific targets is an important future direction, but it should not become a rationale for avoiding the currently underused therapies that already improve outcomes.

## 3 Obesity, Adipose Biology, Muscle, and Liver

### 3.1 Obesity as Multiorgan Disease

The meeting's obesity session offered a broad metabolic synthesis. Obesity is associated with hundreds of complications. The clinically useful insight is that insulin resistance, altered fuel flux, ectopic fat, growth factors, sex hormones, bioactive lipids, extracellular vesicles, and local inflammation provide systemic signals that help explain why diabetes, dyslipidemia, metabolic dysfunction‐associated steatotic liver disease (MASLD), CKD, cardiovascular disease, and cancer cluster. The liver is not simply a passive depot: de novo lipogenesis, impaired fatty‐acid oxidation, and increased VLDL‐triglyceride secretion link hepatic fat to circulating triglycerides and whole‐body insulin resistance (Figure 2).

**FIGURE 2:**
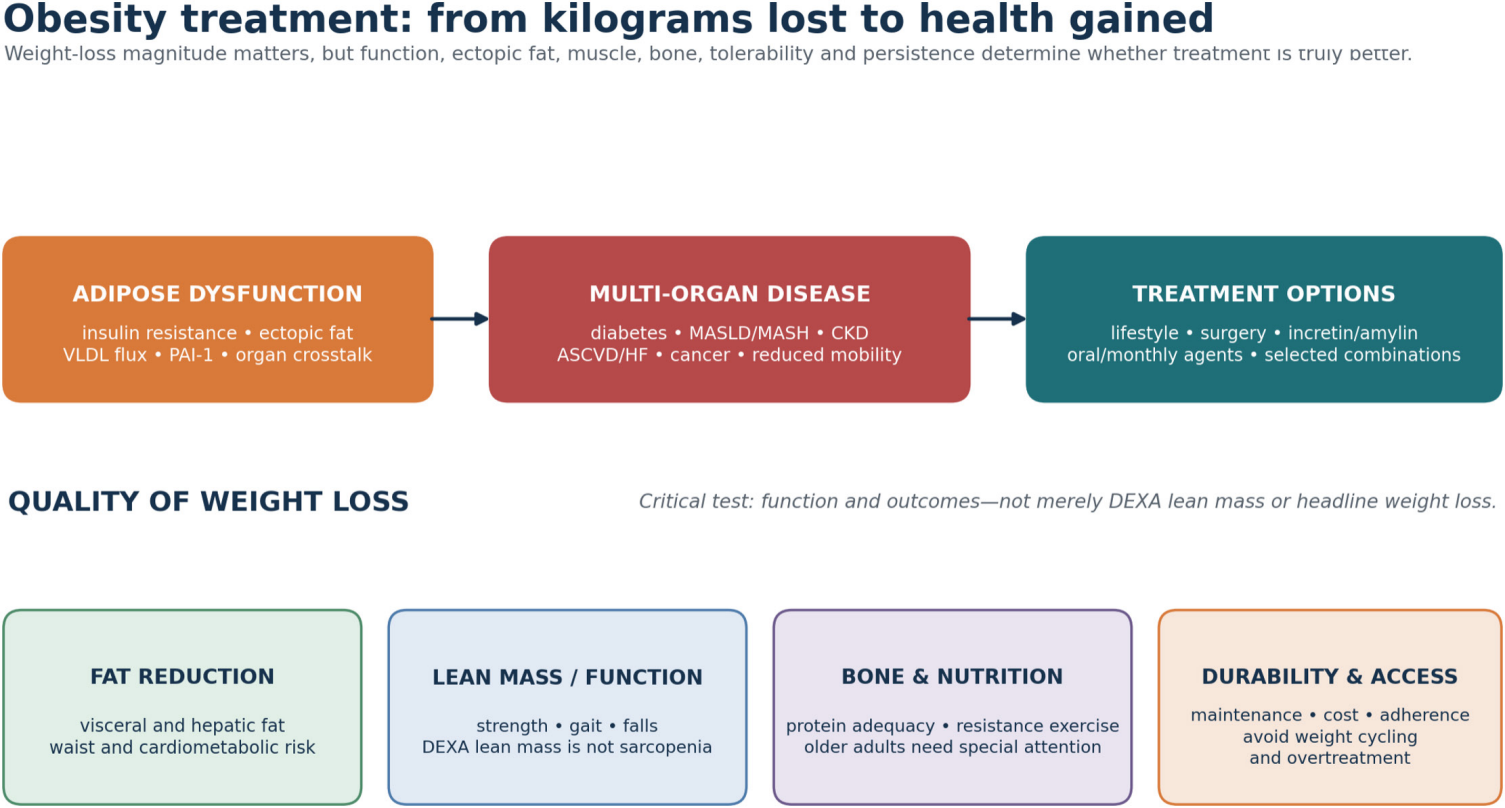
Obesity therapy should be judged by fat distribution, function, bone and muscle health, durability, access, and clinical outcomes—not weight alone.

The simplistic “obesity equals circulating cytokine excess” model must be challenged. In the datasets shown, commonly measured plasma cytokines did not reliably distinguish metabolically healthy from unhealthy obesity, whereas PAI‐1 and small extracellular vesicles were more discriminating. The extracellular‐vesicle data are intriguing because vesicles from metabolically unhealthy obesity impaired insulin signaling and glucose uptake in muscle‐cell models [11]. This is mechanistic evidence, but cannot yet be regarded as a clinical biomarker or therapeutic target.

In a discussion of MASLD, data were presented suggesting that even low alcohol intake may worsen fibrosis risk when metabolic dysfunction is present. The direction is plausible and consistent with the newer MASLD/metabolic dysfunction and alcohol‐associated liver disease framework, but exact “safe” thresholds cannot be inferred from an observational dataset. Counseling should avoid the old assumption that alcohol below a high weekly threshold is metabolically neutral, while still acknowledging measurement error, confounding, and individual risk.

### 3.2 The Quality of Weight Loss

An important concept of the session was the shift from weight‐loss magnitude to weight‐loss quality. Weight reduction inevitably includes fat mass and fat‐free mass. DEXA‐derived lean mass includes water, organs, and nonmuscle tissue; loss of “lean mass” is not synonymous with loss of skeletal muscle, or with weakness or sarcopenia. The clinically relevant endpoints are strength, gait, falls, fractures, ability to perform daily activities, and metabolic function. Body‐composition measurement can be useful, but should not become a surrogate detached from functional outcomes.

Older adults deserve particular caution. Aging is accompanied by loss of muscle, bone, and strength, and repeated stopping and restarting of anti‐obesity medication may produce cycles of rapid fat regain. The Look AHEAD experience showed broad metabolic and quality‐of‐life benefits from lifestyle‐induced weight loss but also a higher rate of frailty fractures in the intervention group [12]. SELECT subgroup imbalances in serious hip/pelvic fractures require attention, but they are not proof that semaglutide causes fractures. The correct response is baseline functional assessment, ongoing counseling to maintain adequate nutrition and resistance exercise, and appropriate monitoring—not avoidance of effective obesity treatment in older adults.

### 3.3 Muscle‐Preserving Agents: Proof of Concept, Not Proof of Better Health

The apitegromab EMBRAZE trial showed approximately 1.9 kg less lean‐mass loss at 24 weeks when a selective myostatin inhibitor was added to tirzepatide, with similar total weight loss [13]. Bimagrumab plus semaglutide also improved body composition in a Phase 2 trial [14]. These studies establish target engagement and DEXA proof of concept. They did not establish fewer falls, better physical function, improved insulin sensitivity, reduced fractures, or longer treatment persistence. In EMBRAZE, exploratory physical‐function outcomes did not show notable between‐group differences, and the trial was small and predominantly female, with the primary analysis using an 80% confidence interval without multiplicity adjustment, so that any findings should be thought of as hypotheses rather than definite clinical evidence.

Premature use of muscle‐preserving biologics should not then be considered a required companion to incretin therapy. Resistance training and adequate dietary protein remain the most evidence‐based approaches to improving strength, and are far less expensive. The problem is effectiveness—getting patients to perform it consistently—and not lack of physiological efficacy. Protein intake should be adequate, but very high protein targets produce only modest additional lean‐mass retention and may not improve insulin sensitivity.

### 3.4 The Anti‐Obesity Drug Pipeline: More Options, Not Automatic Progress

The 2026 landscape includes higher dose injectable semaglutide and oral orforglipron in the United States, as well as rapidly advancing amylin combinations, oral agents, long‐acting agents, and polyagonists [15, 16] This expansion can improve access, manufacturing, patient preference, and maintenance strategies. It also creates a temptation to equate more receptors, higher doses, or monthly dosing with better medicine.

Higher‐dose semaglutide appears to provide incremental rather than transformative efficacy at the highest doses. Oral therapies may be easier to distribute but may have different drug‐interaction, adherence, and off‐target considerations. The combination of the amylin receptor agonist cagrilintide with semaglutide has strong Phase 3 efficacy in T2D, including as add‐on to basal insulin, but it is not clear whether there is definitive head‐to‐head noninferiority in comparison with tirzepatide [17]. Likewise, reported > 30% weight loss with retatrutide may be preliminary; the peer‐reviewed Phase 2 publication reported 24.2% mean loss at 48 weeks at the highest dose [18]. These are examples where “hype” may exceed the evidence.

The induction‐maintenance‐de‐escalation model is clinically more important than the race for the highest percentage loss. SURMOUNT‐MAINTAIN supports continued treatment and shows that dose reduction can preserve more benefit than discontinuation [19]. In practice, cost, tolerability, and access may drive off‐label extended intervals; this is pragmatic but not well validated. A medication that produces slightly less weight loss but is affordable, tolerated, and continued will ultimately outperform a more potent drug that is stopped.

### 3.5 Obesity Treatment in Older Adults

The observation that the BMI‐mortality association attenuates with age should not be interpreted as evidence that obesity is harmless after age 70. Competing illness, reverse causation, and the limitations of BMI complicate those epidemiologic curves. The treatment target in older adults often does shift from longevity alone toward function, symptoms, mobility, sleep apnea, diabetes control, HFpEF, and quality of life. The Lancet Commission's distinction between preclinical and clinical obesity and its movement beyond BMI‐only diagnosis are useful, provided the framework does not create unnecessary testing or deny treatment to people whose risk is real although organ dysfunction is not yet obvious [20].

## 4 Critical Appraisal

**Table:** 

Established/actionable	Emerging/plausible	Needs skepticism
eGFR plus UACR screening; indicated RAAS blockade; SGLT2 inhibitors; finerenone in eligible T2D/CKD; semaglutide in diabetes; potassium and volume monitoring.	Initial combination therapy; non‐diabetic nsMRA indications; endothelin strategies; aldosterone synthase inhibition; biopsy‐informed precision therapy.	Calling a public‐review draft a final guideline; treating projected additive benefits as randomized evidence; assuming every eGFR dip is benign or that larger is always better.
Treat obesity as chronic disease; assess complications; prioritize resistance exercise, adequate nutrition and durable treatment; screen for MASLD/fibrosis when appropriate.	Body‐composition tools, amylin combinations, oral/monthly agents, polyagonists and selected muscle‐preserving adjuncts.	Equating DEXA lean mass with muscle function; assuming more receptors or more weight loss equals better outcomes; presenting conference‐only pipeline results as established.

The most useful direction is therefore not maximal treatment by default, but earlier identification of dominant risks, selection of complementary mechanisms, planned monitoring and attention to function, affordability and persistence. The third commentary turns to cross‐cutting biology, artificial intelligence and digital health, asking whether richer phenotyping can improve implementation without creating a new layer of overdiagnosis and information overload.

## Funding

The authors have nothing to report.

## Conflicts of Interest

The authors declare no conflicts of interest.
